# Apilimod, a candidate anticancer therapeutic, arrests not only PtdIns(3,5)P_2_ but also PtdIns5P synthesis by PIKfyve and induces bafilomycin A1-reversible aberrant endomembrane dilation

**DOI:** 10.1371/journal.pone.0204532

**Published:** 2018-09-21

**Authors:** Diego Sbrissa, Ghassan Naisan, Ognian C. Ikonomov, Assia Shisheva

**Affiliations:** Department of Physiology, Wayne State University School of Medicine, Detroit, Michigan, United States of America; Wright State University, UNITED STATES

## Abstract

PIKfyve, an evolutionarily conserved kinase synthesizing PtdIns5P and PtdIns(3,5)P_2_, is crucial for mammalian cell proliferation and viability. Accordingly, PIKfyve inhibitors are now in clinical trials as anti-cancer drugs. Among those, apilimod is the most promising, yet its potency to inhibit PIKfyve and affect endomembrane homeostasis is only partially characterized. We demonstrate here for the first time that apilimod powerfully inhibited in vitro synthesis of PtdIns5P along with that of PtdIns(3,5)P_2_. HPLC-based resolution of intracellular phosphoinositides (PIs) revealed that apilimod triggered a marked reduction of both lipids in the context of intact cells. Notably, there was also a profound rise in PtdIns3P resulting from arrested PtdIns3P consumption for PtdIns(3,5)P_2_ synthesis. As typical for PIKfyve inhibition and the concomitant PtdIns(3,5)P_2_ reduction, apilimod induced the appearance of dilated endomembrane structures in the form of large translucent cytoplasmic vacuoles. Remarkably, bafilomycin A1 (BafA1) fully reversed the aberrant cell phenotype back to normal and completely precluded the appearance of cytoplasmic vacuoles when added prior to apilimod. Inspection of the PI profiles ruled out restoration of the reduced PtdIns(3,5)P_2_ pool as a molecular mechanism underlying BafA1 rescue. Rather, we found that BafA1 markedly attenuated the PtdIns3P elevation under PIKfyve inhibition. This was accompanied by profoundly decreased endosomal recruitment of fusogenic EEA1. Together, our data demonstrate that apilimod inhibits not only PtdIns(3,5)P_2_ but also PtdIns5P synthesis and that the cytoplasmic vacuolization triggered by the inhibitor is precluded or reversed by BafA1 through a mechanism associated, in part, with reduction in both PtdIns3P levels and EEA1 membrane recruitment.

## Introduction

Seven phosphoinositides (PIs), all regulating critical cellular processes, are produced in mammalian cells [1–5]. They differ by the number and/or position of the phosphate in the inositol headgroup, and are: phosphatidylinositol (PtdIns) 3P, PtdIns4P, PtdIns5P, PtdIns(3,4)P_2_, PtdIns(4,5)P_2_, PtdIns(3,5)P_2_ and PtdIns(3,4,5)P_3_ [1, 2]. Position-specific kinases and phosphatases govern the PI metabolism and are subject to tight intracellular regulation. The phosphoinositide kinase PIKfyve, an evolutionarily conserved and a sole enzyme in mammals, synthesizes two of the seven PIs, *i*.*e*., PtdIns(3,5)P_2_ and PtdIns5P, by phosphorylating the 5-hydroxyl in inositol headgroups of PtdIns3P and PtdIns, respectively [6–10]. The best characterized cellular functions of PIKfyve are linked to regulating various aspects of both degradative and recycling endosomal trafficking, cytoskeletal rearrangement and autophagy [8, 11–14]. The two PIKfyve products appear to be differentially connected to these processes. Thus, PtdIns5P regulates F-actin remodeling and the non-canonical Vps34-independent autophagy whereas PtdIns(3,5)P_2_ controls fusion and fission events in the endosomal system thereby maintaining endomembrane homeostasis [13, 15–17]. Phenotypically, the PIKfyve dysfunction achieved by means of pharmacological inhibition, genetic inactivation or dominant-negative interference, is manifested by the appearance of massive and progressively enlarging cytoplasmic vacuoles in proliferating mammalian cells [15, 18–20]. This phenomenon is due to selective reduction in PtdIns(3,5)P_2_ but not in PtdIns5P, evidenced by complementation experiments with exogenous delivery of either lipid [21]. Under these conditions only PtdIns(3,5)P_2_ is capable of restoring the normal endomembrane morphology. Concordantly, only PIKfyve mutants with disrupted PtdIns(3,5)P_2_, but with intact PtdIns5P, synthesis trigger aberrant cytoplasmic vacuolation [21]. Mechanistic understanding of the defects eliciting the cell vacuolization is still incomplete. Such information is important in light of observations for increased cell toxicity and death in several cancer types through cell vacuolization.

Apilimod (or STA-5326) is a recently described cell permeable small molecule that specifically inhibits PIKfyve at nanomolar range [22]. It has been first discovered in the context of the toll-like receptor-induced IL-12/23 cytokine production and applied to cohorts of patients with various inflammatory diseases [23–26]. Whereas an anti-inflammatory potency of apilimod failed to be established, the clinical trials have found mild side effects and good tolerability of the inhibitor. Selective cytotoxicity of apilimod in B-cell non-Hodgkin lymphoma resulting from powerful inhibition of PIKfyve has been recently reported [27]. A Phase 1 clinical trial with an orally-active apilimod derivative is ongoing in patients with relapsed or refractory B-cell malignancies [28]. Notably, despite this translational breakthrough, the inhibitory potency of apilimod towards PIKfyve catalytic activity is insufficiently explored. In fact, apilimod has been characterized only as a PtdIns(3,5)P_2_-reducing compound with an IC_50_ = 14 nM [22]. Whether apilimod inhibits PIKfyve-dependent PtdIns5P synthesis *in vitro* or in cellular context is unknown.

As in the case with the YM201636 compound, the first PIKfyve inhibitor to be characterized [20, 29], apilimod application to cells and tissues also triggers the characteristic phenotypic changes in the form of multiple cytoplasmic vacuoles [22]. Intriguingly, we have recently established that cell treatment with low doses of bafilomycin A1 (BafA1) phenotypically rescued or completely precluded the appearance of cytoplasmic vacuoles under PIKfyve inhibition with YM201636 [30]. BafA1 is a widely used powerful inhibitor of the vacuolar class H^+^-ATPases (V-ATPase; IC_50_ = 4–400 nM), which blocks acidification of endosomes, lysosomes and phagosomes by arresting proton pumping from the cytosol [31]. Reportedly BafA1 also inhibits endosome-endosome or autophagosome-endosome/lysosome fusion, which may occur independently of the V-ATPase inhibition and, hence, compartment alkalinization [32–36]. Concordantly, deacidification of membrane organelles by weak bases such as chloroquine or NH_4_Cl is ineffective in preventing the vacuolization triggered by PIKfyve inhibition with YM201636, suggesting that BafA1 protects and reverses the aberrant endomembrane dilation by a mechanism that counteracts endosomal fusion [30]. Molecular details of the BafA1 rescue effect remained to be elucidated.

The potential of apilimod to be a powerful therapeutic tool targeting the PIKfyve pathway in cancer requires a more complete characterization of its intracellular effects. In this study we examined if apilimod inhibits both enzymatic activities of PIKfyve. This was enabled by the expertise in our laboratory to detect and quantify cellular levels of PtdIns5P along with those of PtdIns(3,5)P_2_ and the other PIs by HPLC-based inositol headgroup analyses [9, 19, 37], a challenging approach frequently resulting in overlooked PtdIns5P functional contributions. We report here for the first time that apilimod powerfully inhibits both PtdIns5P and PtdIns(3,5)P_2_ synthesis *in vitro* as well as in intact cells. Given that the two PIKfyve inhibitors apilimod and YM201636 differ in their downstream outcomes [38], we explored a plausible BafA1-dependent reversal of apilimod-triggered vacuolization with a focus on the underlying cellular mechanism of the rescue effect. We identified attenuated rise in intracellular PtdIns3P and reduced recruitment of the fusogenic EEA1 protein, rather then mitigated PtdIns(3,5)P_2_ loss, to be key mechanistic determinants associated with BafA1 prevention of cytoplasmic vacuolization.

## Materials and methods

Apilimod {3-methyl-2-[6-(4-morpholinyl)-2-[2-(2-pyridinyl)ethoxy]-4-pyrimidinyl] hydrazone, benzaldehyde}, obtained from Axon Medchem LLC (USA), and YM201636 {[6-amino-N-(3-(4-(4-morpholinyl)-pyrido[3’,2’:4,5]furo[3,2-d]pyrimidin-2-yl)phenyl)-3-pyridinecarboxamide]}, purchased from Symansis NZ (Timaru, New Zealand), were used as recommended by the manufacturers. BafA1 was purchased from Enzo Life Sciences, Inc., USA. Thin layer chromatography (TLC) 20 x 20 cm glass plates (K6 silica gel 60Å, 250 μm layer thickness) and an HPLC 5-micron Partisphere SAX column were from Whatman. Methylamine (40% w/w solution in water), n-propoanol and tetrabutylammonium bisulfate (TBAS) were from Sigma-Aldrich, USA. Glucose- and inositol-free DMEM was prepared in house in sterile distilled deionized water from amino acids and vitamins purchased from Gibco Laboratories (Life Technologies, Inc., USA) or Sigma (Sigma-Aldrich, USA), and inorganic salts from various commercial sources. [γ-^32^P]ATP (6000 Ci/mmol) and *myo*-[2-^3^H]inositol (22.5 Ci/mmol) were from NEN Du-Pont (Boston, MA) and Perkin Elmer (Boston, MA), respectively. Natural soybean PtdIns was from Avanti Polar Lipids, Inc. (USA). Polyclonal anti-PIKfyve antibody was previously described [39]. Goat polyclonal anti-early endosomal antigen 1 (EEA1) antibodies (N-19) were from Santa Cruz Biotechnology (Santa Cruz, CA).

### Cell cultures and treatments

Human embryonic kidney (HEK) 293 and COS7 cells (ATCC, Manassas, VA) or immortalized podocytes prepared from mouse kidney [40] were grown in high-glucose Dulbecco’s modified Eagle’s medium (DMEM, GE Healthcare, USA) supplemented with 10% fetal bovine serum (FBS, Sigma-Aldrich, USA) and 0.5% penicillin/streptomycin antibiotic solution (10,000 units/mL penicillin and 10 μg/ml streptomycin (“complete media). Cells grown to 80–100% confluence were treated with BafA1, apilimod, YM201636 (all dissolved in DMSO vehicle) or vehicle, separately or in combinations at concentrations indicated in the figure legends. Control dishes receiving only DMSO (0.1% or 0.2%) were run in parallel for each experiment.

### Cell transfection and transduction

HEK293 cells were transfected on 22 x 22- mm coverslips with a cDNA construct encoding a pEGFP-2xFYVE domain derived from the PIKfyve sequence [19, 40] by Lipofectamine 3000 transfection reagent (Invitrogen) using 1/3 of the amounts recommended by the manufacturer. Eighteen h post transfection cells were treated with the inhibitors separately or in combinations and then prepared for confocal microscopy. Cells reaching 90–100% confluence were transduced for 30–48 h with recombinant adenoviruses expressing HA-PIKfyve^WT^ and GFP from separate CMV promoters or only GFP as previously described [15]. Cell infection was monitored by the GFP green fluorescence with a Nikon Eclipse TE 200 inverted fluorescence microscope (Nikon Corp., USA).

### Lipid kinase assay and TLC

These were performed following previously published protocols [39]. Briefly, cell lysates from HEK293 cells infected with recombinant adenovirus expressing HA-PIKfyve^WT^/GFP and collected in RIPA buffer (50 mM Tris·HCl buffer, pH 8.0, containing 150 mM NaCl, 1% Nonidet P-40, and 0.5% Na deoxycholate) containing 1 x protease inhibitors mixture (1 mM phenylmethylsulphonylfluoride, 5 μg/ml leupeptin, 5 μg/ml aprotinin, 1 μg/ml pepstatin, and 1 mM benzamidine) (further as RIPA+), were clarified by centrifugation (14,000 g, 15 min, 4°C). Lysates were subjected to immunoprecipitation with anti-PIKfyve antisera (16 h, 4°C) and protein A-sepharose beads that were added during the last 1.5 h of incubation. Beads were washed once with RIPA+ buffer, twice with 50 mM HEPES (pH 7.4), 1 mM EDTA, 150 mM NaCl, three times with 100 mM Tris·HCl (pH 7.5), 500 mM LiCl, twice with 10 mM Tris·HCl (pH 7.5), 100 mM NaCl, 1 mM EDTA and twice with “assay buffer” (25 mM HEPES, pH 7.4, and 120 mM NaCl, 2.5 mM MgCl_2_ and 2.5 mM MnCl_2_). Kinase reactions containing 100 μM PtdIns [sonicated before use in 20 mM HEPES (pH 7.5), 1 mM EDTA] were first preincubated for 15 min (37°C) in the presence of varying concentrations of BafA1 or apilimod (delivered at a 0.1% DMSO final concentration) or only vehicle (0.1% final concentration). The kinase assay was carried out for 15 min at 37°C with 15 μM ATP and [γ-^32^P]ATP (30 μCi) in a 50 ul volume and terminated with 200 μl 1N HCl. Lipids were then extracted with chloroform, spotted on a TLC glass plate, and resolved by a chromatographic solvent system of n-propanol/2 M acetic acid (65:35 v:v). Generated radioactive products were detected by autoradiography and quantified with ImageJ software (NIH, USA).

### Myo-[2-^3^H]inositol cell labeling and HPLC

Cells were labeled with *myo*-[2-^3^H]inositol following our previous protocols [19, 37, 41], Briefly, cells (in 35 mm dishes) were maintained for 24 h in “starvation” medium (glucose- and inositol-free DMEM, containing 10% dialyzed FBS, 5 μg/ml each of insulin and transferrin, 2 mM pyruvate, 25 mM HEPES (pH 7.4), 50 units/ml penicillin, and 50 μg/ml streptomycin), in a 37°C incubator under a humidified, 5% CO_2_ atmosphere. The medium was then replaced with fresh starvation medium supplemented with 25 μCi/ml *myo*-[2-^3^H]inositol (corresponding to ~1.5 μM final concentration of *myo-*inositol) and cells were labeled for 25 h. Cell treatments were performed in the same labeling medium for 1–2 additional hours as specified in the figure legends. The incubation time was sufficient to reach a condition of 90–100% isotopic equilibrium (i.e., steady-state) across the lipid pools as calculated from the PIP/PI ratios in both HEK293 cells and podocytes as described previously [37, 40, 42]. Cells were washed 3 times in PBS carrying phosphatase inhibitors (50 mM NaF, 10 mM Na pyrophosphate, 25 mM Na β-glycerophosphate, and 2 mM Na metavanadate) and lipids were extracted with TBAS reagent [CH_3_OH/1M HCl (1:1 v:v)] in the presence of 5 mM EDTA and 5 mM tetrabutylammonium sulfate], deacylated at 54°C for 60 min with methylamine reagent (6.4 ml methanol, 3 ml 40% w/w methylamine, 1.3 ml water, 1.1 ml n-butanol, with 1 mM Na EDTA, pH 8). Glycerophosphoinositol phosphates (GroPInsP) were analyzed by HPLC (Waters 5215) on a 5-micron Partisphere SAX column eluted as previously described [19, 37, 39, 41, 43]. Fractions were collected every 0.25 min and analyzed for [^3^H] radioactivity after the addition of scintillation mixture. Data evaluation and documentation was performed by Microsoft Excel. Individual peak radioactivity was quantified by area integration and presented as a percentage of the summed radioactivity from the [^3^H]GroPIns3P, -4P, -5P, -(3,5)P_2_, and -(4,5)P_2_ peaks (“total radioactivity”).

### Light and fluorescence microscopy

Live HEK293 cells on 60 mm dishes were viewed for induction and progression of the vacuolation phenotype with a Nikon Eclipse TE 200 inverted fluorescence microscope. The phase-contrast images were captured by the differential interference contrast (DIC) with 40x objective and a SPOT RT Slider CCD camera (Diagnostic Instruments) as previously described [30]. Cells having at least 7–8 perinuclear translucent vacuoles were considered as vacuolated. Under each experimental design, 200 cells/condition from different fields were counted in 3 separate experiments. HEK293 cells on glass coverslips expressing eGFP-2xFYVE^PIKfyve^ were fixed with 3% paraformaldehyde for 30 min and then visualized by confocal microscope. For EEA1 immunofluorescence microscopy, HEK293 cells, seeded on glass coverslips were fixed, permeabilized with saponin and then stained with anti-EEA1 as described [44]. Anti-EEA1 was visualized by FITC-conjugated donkey anti-goat IgG (Jackson Immuno Research Laboratories). Coverslips, mounted on slides were observed by a motorized inverted confocal microscope Olympus IX81 using 40x or 60x water immersion objectives. GFP or FITC signals were captured by a standard green fluorescence filter. Images were taken by a Hamamatsu Orca Flash4.0 digital CCD camera.

### Other methods

Protein concentration was determined with a bicinchoninic acid protein assay kit (Pierce, USA). Lipid levels were quantified from scanned images for the intensities of the TLC spots on autoradiograms taken with an Epson Perfection V700 Photo flatbed scanner (Epson) using ImageJ software (NIH). Several films of different exposure times were quantified to ensure the signals were within the linear range. Half maximal inhibitory concentration (IC_50_) values were estimated in GraphPad Prism 6.0 (GraphPad Software, USA) using the variable slope non-linear regression curve fitting option. ImageJ software (NIH) was used to quantify cell fluorescence in 30–100 randomly selected transected of nontransfected cells in each condition. A region close to each of the evaluated cells was selected to establish the background fluorescence that was subtracted from the value of the cell fluorescence. The corrected cell fluorescence was then expressed as integrated density and related to the integrated density in controls.

### Statistics

Results are presented as mean ± standard error (SEM). Significant differences between treated vs. control sample data were evaluated by the Student’s t-test for independent samples and a one-tail t-test for paired samples. More than 2 experimental groups were analyzed by one-way analysis of variance (Anova) and Tukey multiple comparison test. *P*-values < 0.05 was considered statistically significant.

## Results

### Apilimod inhibits in vitro PIKfyve-catalyzed synthesis of PtdIns5P along with that of PtdIns(3,5)P_2_

**Fig 1:**
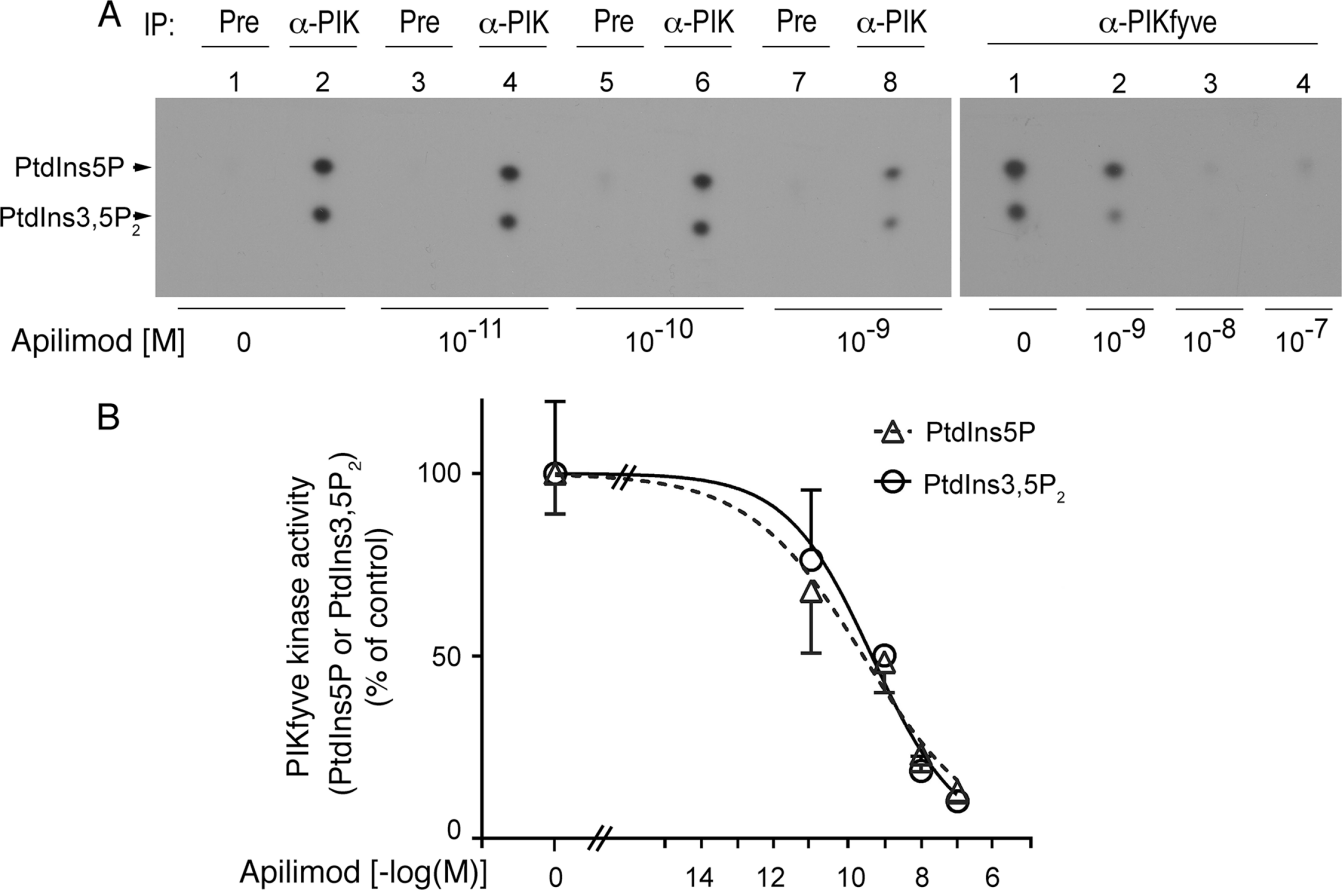
Apilimod inhibits not only PIKfyve-catalyzed synthesis of PtdIns(3,5)P_2_ but also that of PtdIns5P. Lysates, derived from HEK293 cells transduced with recombinant adenovirus expressing HA-PIKfyve^WT^ and GFP from separate promoters, were immunoprecipitated with a polyclonal anti-PIKfyve (α-PIK) or preimmune (Pre) sera. Washed immunoprecipitates (IPs) were pretreated with various apilimod concentrations or with vehicle alone (0.1% DMSO) for 15 min at 37°C along with PI substrate and then subjected to a lipid kinase assay with 15 μM ATP and [γ-^32^P]ATP (30 μCi) in a 50-μl final volume. Lipid products were resolved by TLC n-propanol/2 M acetic acid solvent system (65:35 v:v). (A): Shown are autoradiograms from representative TLCs out of 6 independent experiments demonstrating that both PIKfyve lipid products, i.e., PtdIns5P and PtdIns(3,5)P_2_ (denoted by arrowheads) are inhibited significantly at low nanomolar concentrations of apilimod. (B): Quantification of the autoradiograms from six experiments using variable slope non-linear regression curve fitting option of ImageJ software (mean ± SEM). Note that the two lipids are inhibited with a similar efficiency.

Apilimod-dependent inhibition of the PIKfyve-catalyzed PtdIns5P synthesis *in vitro* has not been tested in the original study characterizing the drug as a PIKfyve inhibitor [22]. Additionally, a recent report that did examine a perceived PtdIns5P reduction by apilimod using a cell-free microfluidic enzyme assay and a synthetic di-C6 PI substrate yielded a negative result [38]. To address this paucity, we performed a traditional *in vitro* lipid kinase activity assay using radiolabeled ATP, a native enzyme substrate and PIKfyve, immunopurified from HEK293 cells. Subsequent to short preincubation (15 min at 37°C) with different concentrations of apilimod (0–100 nM), the kinase reaction was conducted for 15 min in the presence of [γ -^32^P]ATP and a native PtdIns substrate from soybean, which supports production of both PtdIns5P and PtdIns(3,5)P_2_ as we previously established [9, 10, 21, 37, 39, 45, 46]. The lipid products were resolved by TLC with the n-propanol/acetic acid, rather than the basic organic solvent system, as the former avoids comigrating unspecific components yet provides a clear-cut separation of PtdIns(3,5)P_2_ from PtdIns5P as we detailed elsewhere [29]. Strikingly, we observed that apilimod at low nanomolar concentrations powerfully inhibited not only PtdIns(3,5)P_2_ synthesis but also that of PtdIns5P (Fig 1A). Quantification of the radioactive spots for the two lipid products from 6 independent experiments determined IC_50_ values of ~0.4 nM for either PtdIns(3,5)P_2_ or PtdIns5P production (Fig 1B). Our estimated IC_50_ value for inhibition of PtdIns(3,5)P_2_ is substantially lower than those reported previously, *i*.*e*., 14 nM in Ref. [22] and 5 nM in Ref. [38]. The discrepancy is likely related to the different or suboptimal conditions of the *in vitro* assays in those studies [22, 38], including non-physiological synthetic substrates with short (C6) acyl chains, the PIKfyve source, incubation times, reaction buffers, methods of detection, etc. Moreover, the IC_50_ value in Ref. [22] seems to be at odds with the nearly complete inhibition of PtdIns(3,5)P_2_ in intact cells at apilimod doses as low as 10 nM, as reported in the same study. Irrespectively, our data unequivocally demonstrate that apilimod is a powerful inhibitor of the PIKfyve-catalyzed *in vitro* synthesis of both PtdIns(3,5)P_2_ and PtdIns5P.

### Apilimod reduces not only PtdIns(3,5)P_2_ production but also that of PtdIns5P in intact cells

**Fig 2:**
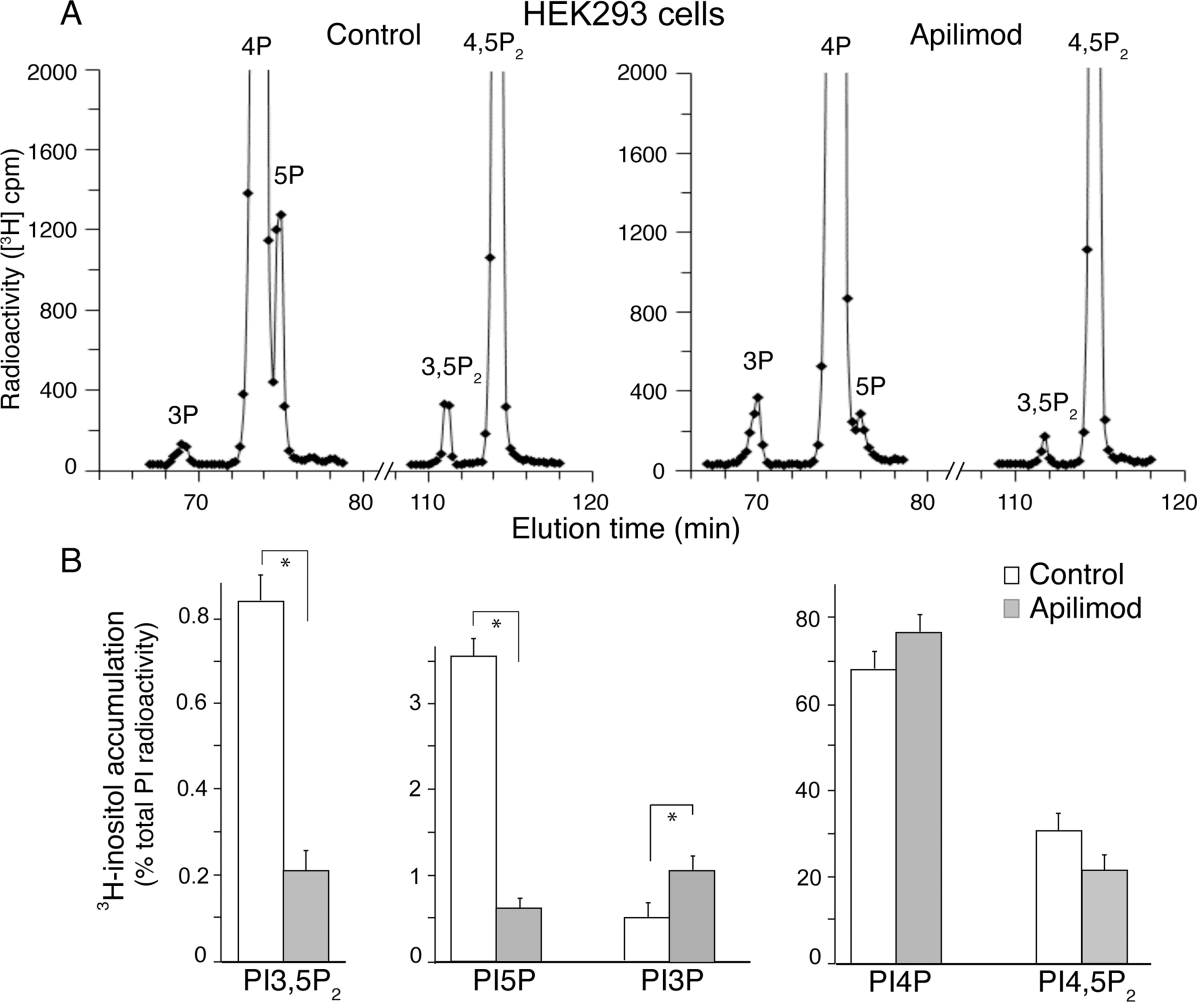
In intact HEK293 cells apilimod reduces not only PtdIns(3,5)P_2_ but also PtdIns5P. HEK293 cells cultured in complete media and grown to 90–100% confluence were incubated for 24 h at 37°C in glucose- and inositol-free “starvation” medium prior to labeling for 25 h with 25 μCi/ml *myo*-[2-^3^H]inositol. Cells were then treated with vehicle (control, 0.1% DMSO) or 100 nM apilimod for 60 min at 37°C in the same labeling medium prior to lipid extraction, deacylation and HPLC separation of deacylated GroPIns. Fractions were analyzed for [^3^H] radioactivity. (A): Representative HPLC [^3^H]GroPInsP profiles from control (left panels) and apilimod treated (right panels) HEK293 cells showing the large reduction in PtdIns5P or PtdIns(3,5)P_2_ as well as a significant rise in PtdIns3P induced by apilimod. (B): Quantification of apilimod-induced changes in PtdIns3P, PtdIns4P, PtdIns5P, PtdIns(3,5)P_2_ and PtdIns(4,5)P_2_ levels from 3 independent experiments (mean ± SEM) (*), *P*<0.05.

Quantitation of intracellular PtdIns5P by HPLC inositol headgroup analyses is challenging because, first, PtdIns5P represents only a minor fraction of the radiolabeled PI pool and, second, its HPLC elution characteristics are very similar to those of the abundant PtdIns4P. These obstacles have rendered PtdIns5P undetectable under conventional HPLC runs [47]. Optimized conditions in the HPLC elution have made the chromatographic separation of PtdIns5P detectable [9, 19, 37, 39, 43, 47]. Despite this, cellular PtdIns5P remained unmeasured or undetected in recent reports characterizing apilimod inhibitory potency in HeLa or RAW264.7 cell lines by PI separation with HPLC [22, 48]. Furthermore, a study applying an alternative method for detecting intracellular PtdIns5P (i.e., the mass assay, [49]) demonstrated that basal PtdIns5P is refractory to apilimod treatment in cardiomyoblasts [50]. To reveal if apilimod inhibits PtdIns5P production in a cellular context we performed metabolic labeling of HEK293 cells with *myo-*[2-^3^H]inositol and examined the PI profiles by our well-characterized protocols for HPLC-based inositol headgroup separation, attaining a clear-cut detection and quantification of PtdIns5P along with PtdIns(3,5)P_2_ and the other PIs [19, 30, 37, 39, 43]. Subsequent to labeling and prior to lipid extraction/deacylation, cells were treated with 100 nM apilimod for 60 min. As illustrated in Fig 2A and 2B, the HPLC profiles and the quantitation of the radioactive PI peaks by pair-wise comparison unequivocally demonstrated that apilimod markedly reduced not only steady-state levels of PtdIns(3,5)P_2_ but also those of PtdIns5P (by 4–5 fold). Steady-state levels of PtdIns(4,5)P_2_ and PtdIns4P were insignificantly changed, whereas those of PtdIns3P were increased by ~2-fold (Fig 2A and 2B).

A rise in PtdIns3P is a typical response under perturbed PIKfyve enzymatic activity, which is observed in different cell types, including RAW macrophages, adipocytes, MEFs, HEK293 and HeLa, metabolically labeled with *myo*-[2-^3^H]inositol under a protocol similar to the one used herein [10, 19, 22, 37, 48]. It results from arrested PtdIns3P consumption for PtdIns(3,5)P_2_ production and unperturbed turnover of residual PtdIns(3,5)P_2_ to PtdIns3P by the Sac3 phosphatase [10]. Consistent with data presented in Fig 2, in the above-cited studies the decreases in PtdIns(3,5)P_2_ also exceed the increases in PtdIns3P by 1.5–2.5-fold. We suggest that this ~2-fold difference of the opposite changes in PtdIns(3,5)P_2_ vs. PtdIns3P under the PIKfyve activity arrest is most likely related to the fact that steady-state levels of PtdIns3P exceed those of PtdIns(3,5)P_2_. Additionally, it is also possible that the rate of PtdIns(3,5)P_2_ (and likely, of PtdIns5P) turnover is higher compared to the rate of PtdIns3P synthesis. This latter mechanism might be particularly relevant in cells (i.e., podocytes, see further), in which steady-state levels of PtdIns3P exceed those of PtdIns(3,5)P_2_ by nearly 10-fold, thus making the conversion of the latter to PtdIns3P insufficient to account for the 2-fold rise in PtdIns3P under PIKfyve inhibition. In any case, the data in Fig 2 illustrate that apilimod inhibits synthesis of PtdIns5P along with that of PtdIns(3,5)P_2_.

**Fig 3:**
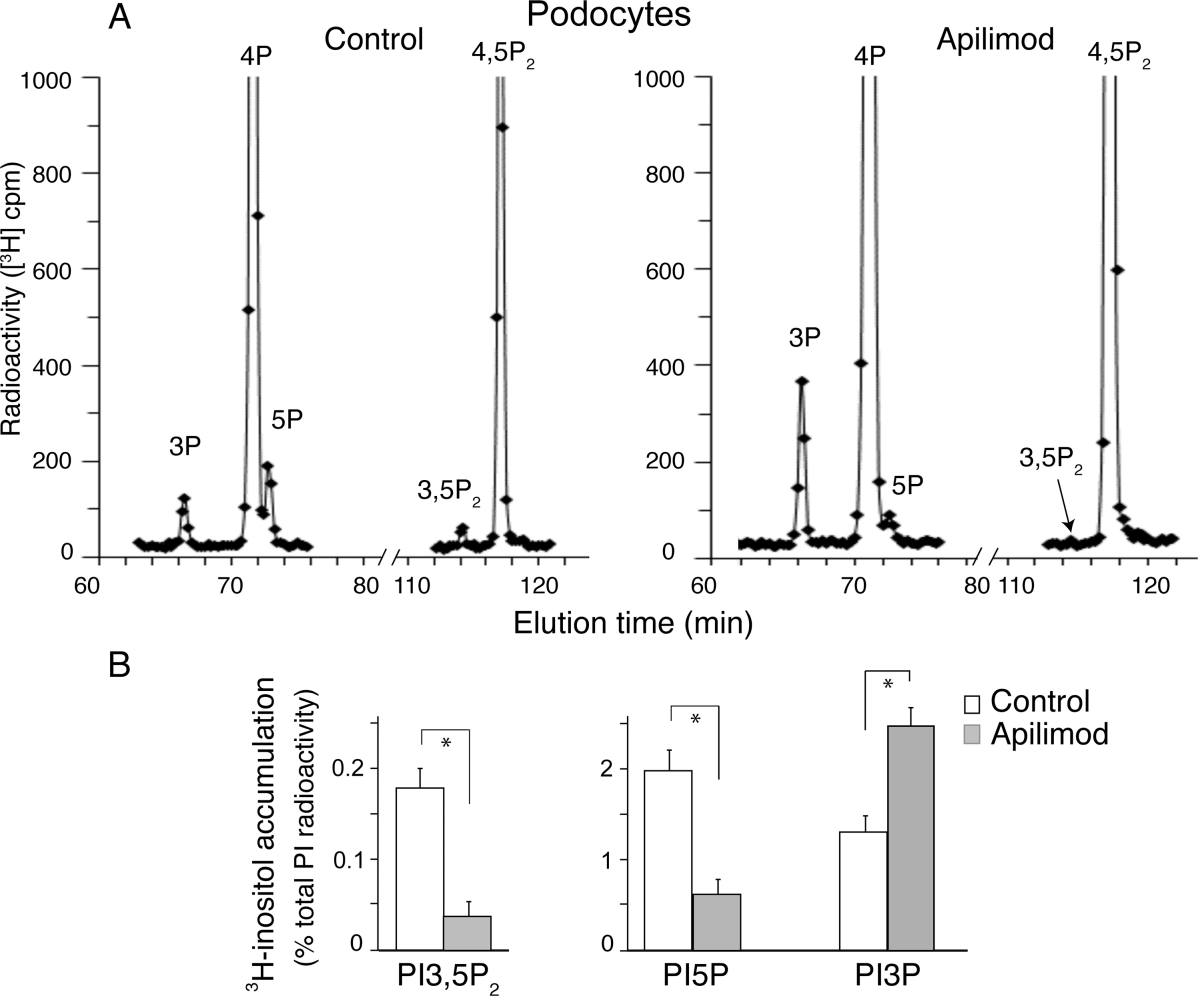
In intact podocytes apilimod reduces not only PtdIns(3,5)P_2_ but also PtdIns5P. Podocytes cultured in complete media and grown to 90–100% confluence were labeled as described under Fig 2. Cells were then treated with vehicle (control, 0.1% DMSO) or 100 nM apilimod for 60 min at 37°C in the same labeling medium prior to lipid extraction, deacylation and HPLC separation of deacylated GroPIns. Fractions were analyzed for [^3^H] radioactivity. (A): Representative HPLC [^3^H]GroPInsP profiles from control (left panels) and apilimod treated (right panels) podocytes, showing the large reduction in PtdIns5P or PtdIns(3,5)P_2_ as well as a significant rise in PtdIns3P induced by apilimod. (B): Quantification of apilimod-induced changes in PtdIns3P, PtdIns4P, PtdIns5P, PtdIns(3,5)P_2_ and PtdIns(4,5)P_2_ levels from 3 independent experiments (mean ± SEM) (*), *P*<0.05.

To reveal whether this powerful reduction of PtdIns5P, along with PtdIns(3,5)P_2_, could be reproduced in other cell types, we subjected an immortalized podocyte cell line derived from mouse kidney [40] to a similar analysis. Following *myo*-[2-^3^H]inositol metabolic labeling, podocytes were treated for 40 min with apilimod (100 nM), when lipids were extracted, deacylated and analyzed by HPLC. Similar to our results in HEK293 cells we observed that apilimod treatment markedly reduced steady-state levels of PtdIns5P, along with those of PtdIns(3,5)P_2_ (by 4-5-fold, Fig 3A and 3B). The typical rise in PtdIns3P levels under inhibited PtdIns(3,5)P_2_ production was also apparent in this cell type, being nearly 2-fold over PtdIns3P in control podocytes. Taken together our data in intact HEK293 cells and mouse podocytes clearly demonstrate that apilimod is a powerful inhibitor of both PtdIns(3,5)P_2_ and PtdIns5P production.

### Apilimod-triggered cytoplasmic vacuolation is precluded by BafA1

**Fig 4:**
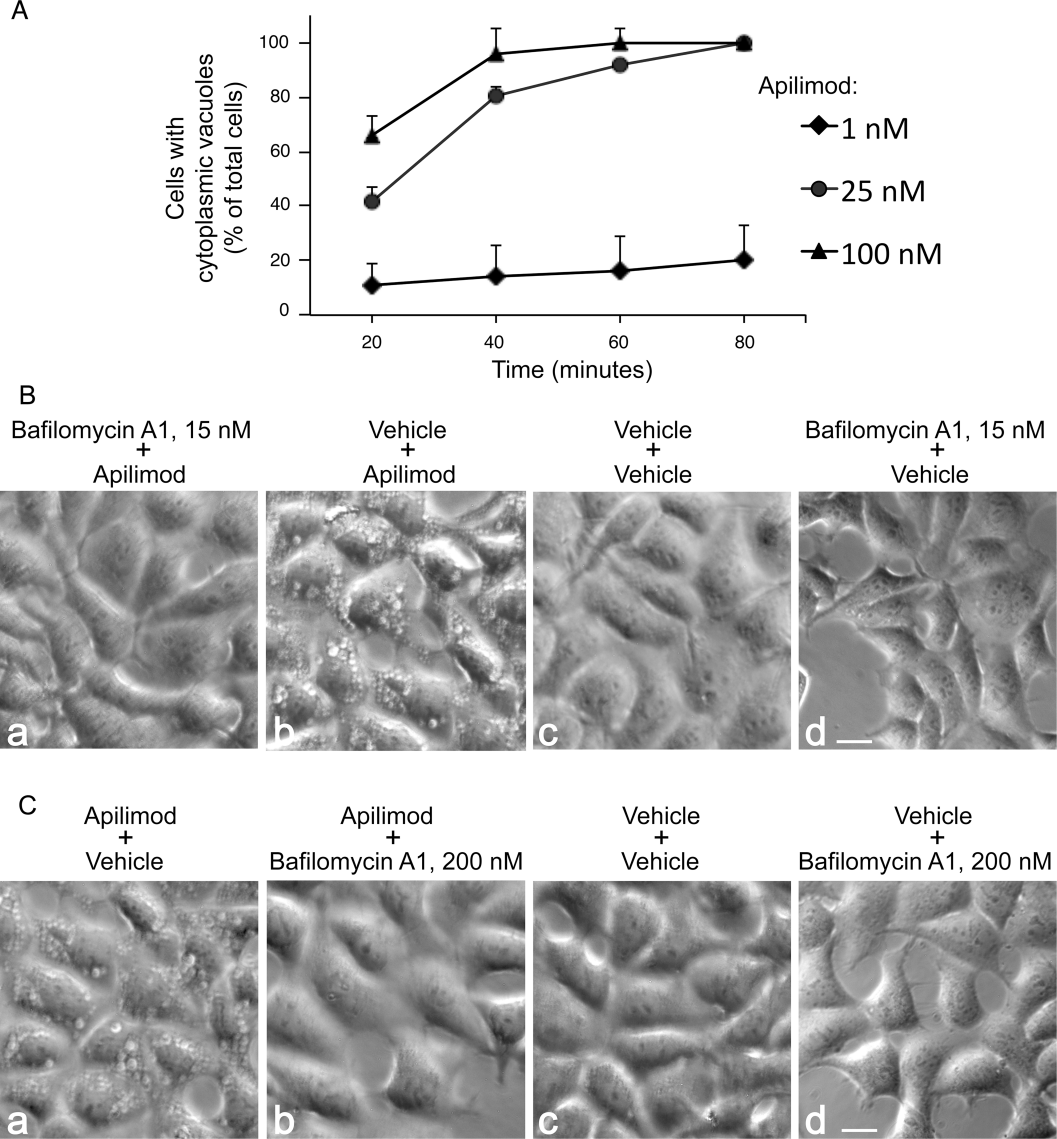
Apilimod-triggered cytoplasmic vacuoles are prevented or dissipated by BafA1 treatment. (A): HEK293 cells grown to 70–80% confluence in complete DMEM medium were treated with various concentrations of apilimod in DMSO (0.1% final concentration) for 1–80 min at 37°C prior to monitoring vacuolation extent by light microscopy. Shown is a quantitative analysis of vacuolation responses, presented as percentage of the total cells, determined by counting at least 200 cells/condition from 10 or more random fields in 4 separate experiments (mean ± SEM). (B): HEK293 cells first pretreated with BafA1 (15 nM) or DMSO (0.1%) for 40 min at 37°C prior to further addition of apilimod (100 nM) or DMSO (0.1%) for 60 min. BafA1 precluded the appearance of any vacuoles. (C): HEK293 cells were pretreated with apilimod (100 nM) or DMSO (0.1%) for 60 min at 37°C. BafA1 (200 nM) or the DMSO vehicle (0.1%) was included for an additional 90 min. BafA1 dissipated apilimod-induced multiple vacuoles. (B and C): Presented are typical phase-contrast images of live cells out of 4 independent experiments with similar result. In each experiment at least 200 cells/condition from several random fields were inspected. Lack of vacuoles upon BafA1 treatment before or after apilimod was seen in ~98% of the monitored cells in each experiment. Bar, 10 μm.

A phenotypic hallmark of PIKfyve perturbation is the formation of multiple cytosolic vacuoles as identified by us and confirmed by other investigators [8, 15, 18]. Consistent with apilimod being a powerful PIKfyve inhibitor, treatment of RAW264.7 cells with 10–100 nM apilimod for a period of 60–180 min reportedly triggers appearance of vacuoles [22, 48]. To address the time and dose dependence of the apilimod-triggered vacuolation in HEK293 cells used in this study, we treated cells with 1–100 nM apilimod for 0–80 min. As illustrated in Fig 4A, vacuolization was apparent after 20 min of incubation with 25 or 100 nM of apilimod. Vacuoles were observed in nearly all cells after 40 min treatment with 100 nM apilimod and persisted until the end of the indicated period (Fig 4A) and beyond (35 h). Under low concentrations of apilimod (1 nM) vacuoles appeared in 15% of the cells after an extended incubation period (80 min; Fig 4A). Noteworthy, the baseline degree of vacuolation in apilimod-untreated cells was practically zero as apparent from the images of control cells, illustrated in Fig 4B and 4C, panels c.

We have previously established that short pretreatment of COS7 cells with low doses of BafA1 (5–15 nM) completely precluded the appearance of cytoplasmic vacuoles under PIKfyve inhibition by YM201636 [30]. Likewise, COS7 cells that exhibited vacuoles due to YM201636 treatment were phenotypically rescued upon subsequent addition of higher doses of BafA1 (200 nM; 80 min) [30]. To reveal if BafA1 could similarly prevent the formation of, and/or rescue already formed vacuoles under PIKfyve inhibition with apilimod, we first pretreated HEK293 cells with BafA1 (15 nM, 40 min) and then added apilimod (100 nM for 60 min). As illustrated in Fig 4B, even with a relatively high dose of apilimod (100 nM), BafA1 completely precluded the appearance of cytoplasmic vacuoles. The latter were not seen for as long as 2–24 h post-treatment with apilimod. Similarly, higher doses of BafA1 (200 nM; 1.5 h) completely reversed the aberrant vacuolation phenotype back to normal morphology in HEK293 cells treated with 100 nM apilimod for 60 min (Fig 4C). Control treatments with vehicle with or without BafA1 did not alter the normal cell morphology (Fig 4B and 4C). Taken together, these data indicate that, as with the YM201636 inhibitor, BafA1 prevents and reverses apilimod-triggered aberrant cytoplasmic vacuolation.

### BafA1 rescue is associated with suppressed PtdIns3P elevation rather than restored PtdIns(3,5)P_2_ loss

**Fig 5:**
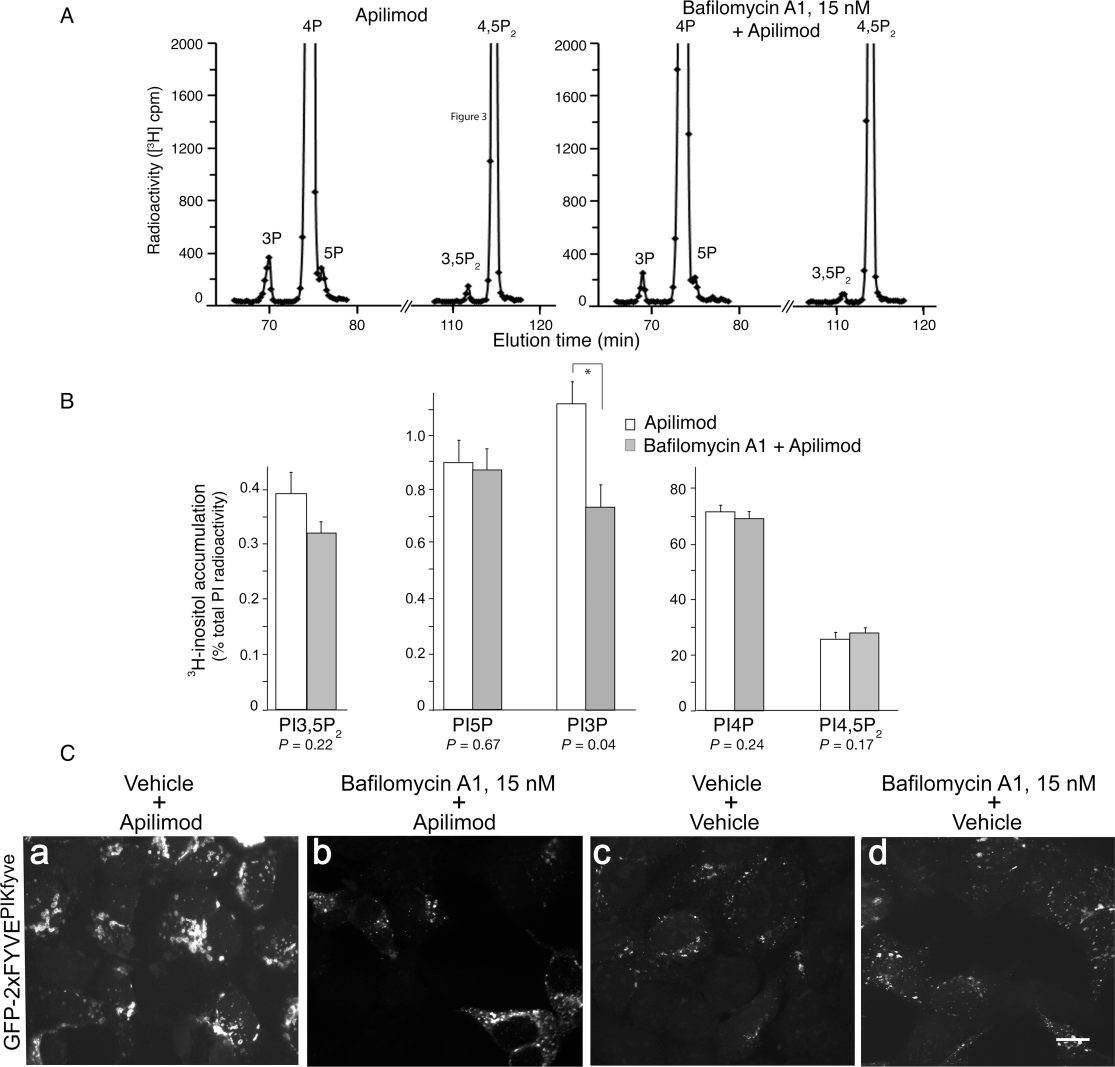
BafA1 suppresses PtdIns3P elevation but does not mitigate PtdIns(3,5)P_2_ reduced by apilimod. (A and B): HEK293 cells, cultured in complete media and grown to 90–100% confluence, were incubated for 24 h at 37°C in “starvation” medium prior to labeling with *myo*-[2-^3^H]inositol as described under Fig 2. Cells were treated at 37°C for 40 min with vehicle (control, 0.1% DMSO) or BafA1 (15 nM) followed by 100 nM apilimod (in DMSO) or vehicle (0.1% DMSO) for an additional 60 min in the same labeling medium. Lipids were extracted, deacylated and GroPIns were separated by HPLC. Shown are representative HPLC [^3^H]GroPInsP profiles from apilimod (left panel) and BafA1+apilimod treated HEK293 cells (right panel) (A) and quantification of BafA1-induced changes in PtdIns3P, PtdIns4P, PtdIns5P, PtdIns(3,5)P_2_ and PtdIns(4,5)P_2_ levels from 3 independent experiments (mean ± SEM), (*), *P*<0.05 (B). Note that BafA1 reduces the PtdIns3P elevation by apilimod without ameliorating reduced PtdIns(3,5)P_2_ levels. (C): Confocal microscopy analysis in transfected HEK293 cells expressing PtdIns3P-binding reporter GFP-2xFYVE^PIKfyve^ at low levels. Fluorescence signals associated with GFP-2xFYVE are markedly increased in cells with apilimod (panels a vs. c) and drastically reduced by BafA1 pretreatment (panels a vs. b), resembling those in transfected control cells receiving only vehicle (panel c) or only BafA1 (panel d). Shown are typical confocal images (60x objective) out of inspected 100 transfected cells/condition from several randomly selected fields. Bar, 10 μm.

We have previously observed that the cytoplasmic vacuolization under perturbed PIKfyve activity in several proliferating mammalian cells occurs only if steady-state levels of PtdIns(3,5)P_2_ are markedly reduced, *i*.*e*., >2-fold below those of controls [19, 30]. Therefore, plausible direct or indirect interference of BafA1 on either PIKfyve, PIKfyve regulatory proteins ArPIKfyve/Sac3 [8, 18] or other yet-to-be-identified PIKfyve regulators, to alleviate the PIKfyve inhibition, restore cellular PtdIns(3,5)P_2_ and, thus, prevent cytoplasmic vacuolization, is conceivable. To address this potential mechanism for BafA1 prevention of the apilimod-triggered aberrant phenotype, we inspected the PI profiles by HPLC inositol headgroup analyses in HEK293 cells metabolically labeled with *myo*-[2-^3^H]inositol. At the end of the labeling period, cells were treated with BafA1 (15 nM) or vehicle for 40 min prior to apilimod (100 nM) or vehicle addition for 60 min. Under these conditions, BafA1 completely precluded the appearance of cytoplasmic vacuoles (see Fig 4B). Quantitation of PI changes revealed that under this 2-step protocol, apilimod treatment reduced PtdIns5P/PtdIns(3,5)P_2_ and elevated PtdIns3P vs. control (only DMSO) at a level, very similar to that shown in Fig 2. Intriguingly, we observed that steady-state levels of PtdIns(3,5)P_2_ not only remained reduced by BafA1 pretreatment but even exhibited a trend, although not statistically significant, for a further decrease (Fig 5A and 5B). Likewise, BafA1 pretreatment did not cause significant changes in steady-state levels of PtdIns(4,5)P_2_, PtdIns4P and PtdIns5P compared to cells that received only apilimod (Fig 5A and 5B). Strikingly however, under these conditions we observed that BafA1 precluded the apilimod-dependent ~2-fold rise in intracellular [^3^H]PtdIns3P levels vs. control (Fig 5A and 5B). Quantitation by pair-wise comparison of the PI radioactive peaks from three independent experiments demonstrated that steady-state levels of PtdIns3P in BafA1 pretreated/apilimod treated cells were ~1.7-fold lower compared to those in cells treated only with apilimod + vehicle (Fig 5B). In fact, steady-state levels of PtdIn3P in BafA1 pretreated/apilimod treated cells were reduced to nearly control levels seen in cells only with vehicle (0.74% vs. 0.64; *P* = 0.47, n = 3; Fig 5B and S1 Fig). Importantly, HEK293 cells treated only with BafA1 (15 nM, 100 min) + vehicle (last 60 min) showed unaltered steady-state levels of PtdIns3P vs. those found in control cells that received only vehicle (0.61% vs. 0.64%, mean, ± SEM; *P* = 0.89, n = 3; S1 Fig). Consistent with our observations with BafA1, PtdIns3P levels also remained unaltered by slight alkalinization via treatment with weak bases such as concanavalin A1 or NH_4_Cl of RAW macrophages [51]. Together, this data indicate that BafA1 attenuated only elevated PtdIns3P by apilimod treatment but did not affect the basal PtdIns3P in untreated cells.

Concordantly, fluorescence microscopy analyses with an intracellular PtdIns3P reporter, eGFP-2xFYVE^PIKfyve^, that binds PtdIns3P-enriched endosome membranes with high affinity [19, 40], revealed marked increases and decreases in membrane-associated fluorescence intensity by vehicle/apilimod- and BafA1/apilimod treatments, respectively (Fig 5C). Of note, in order to avoid high overexpression of the eGFP-2xFYVE biosensor, the transfection experiments were performed at very low doses of both cDNA and lipofectamine. Altogether, these data indicated that the presumed BafA1-dependent alleviation of reduced PtdIns(3,5)P_2_ levels was not the mechanism underlying abrogated cytoplasmic vacuolization. Rather, we found that BafA1 pretreatment abolished the characteristic PtdIns3P rise seen under PIKfyve inhibition with apilimod.

### BafA1 also attenuates PtdIns3P rise under YM201636-inhibited PIKfyve

**Fig 6:**
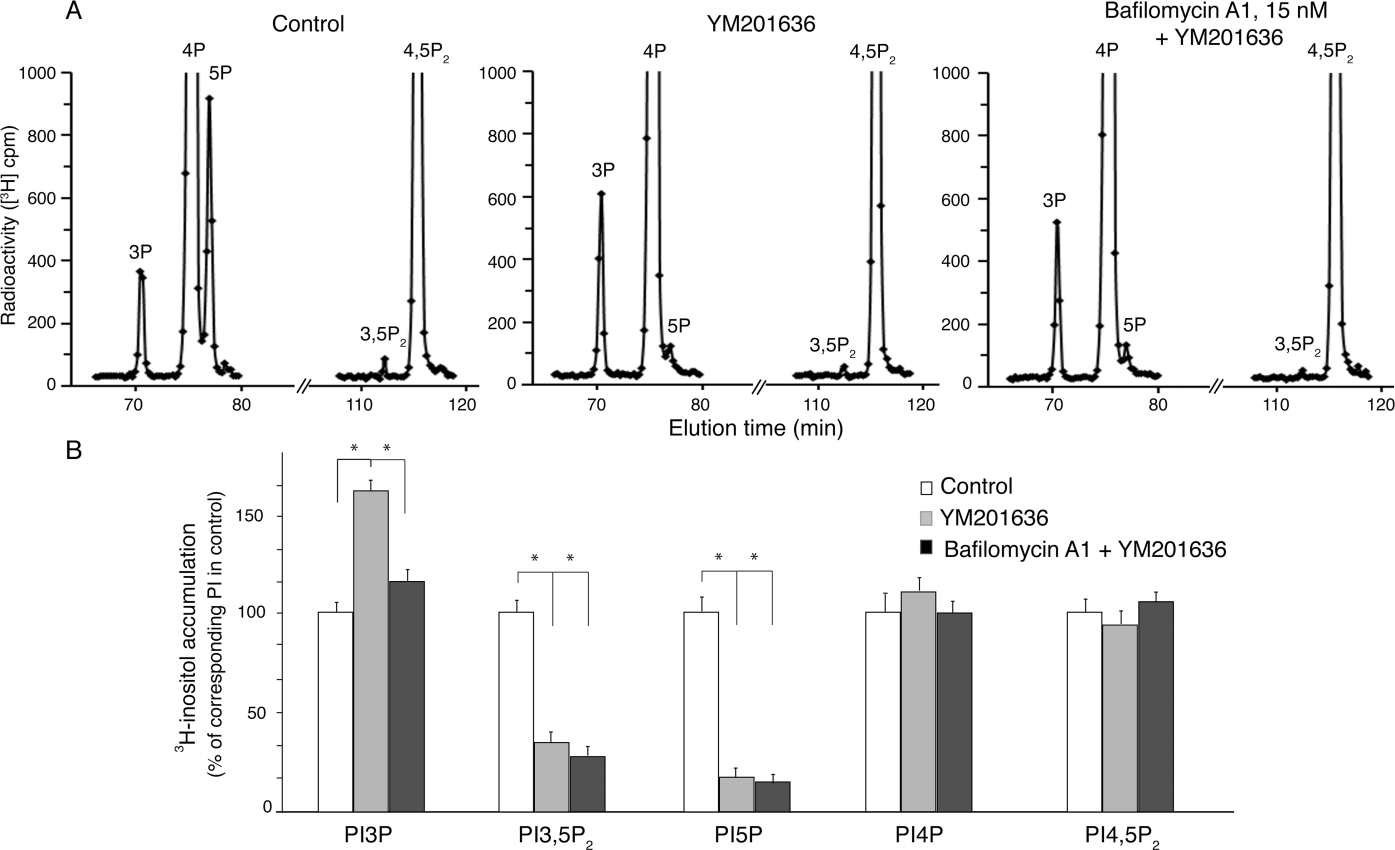
BafA1 attenuates PtdIns3P elevation but does not ameliorate PtdIns(3,5)P_2_ reduced by YM201636. HEK293 cells cultured in complete media grown to 90–100% confluence were labeled with *my*o-[2-^3^H]inositol as described under Fig 2. Cells were then treated with vehicle (control, DMSO) or BafA1 (15 nM; in DMSO) for 40 min at 37°C when YM201636 (800 nM; in DMSO) or vehicle was added for an additional 60 min in the same labeling medium. Lipids were extracted and deacylated, followed by HPLC separation of deacylated GroPIns. (A): Shown are representative HPLC [^3^H]GroPInsP profiles from control (left panel), YM201636- (middle panel) and BafA1+YM201636-treated cells (right panel), demonstrating that BafA1 arrested PtdIns3P elevation induced by YM201636. (B): Quantification of YM201636- or BafA1-dependent changes in PtdIns3P, PtdIns4P, PtdIns5P, PtdIns(3,5)P_2_ and PtdIns(4,5)P_2_ from three independent experiments, presented as a percent of the corresponding control (mean ± SEM) and analyzed by one-way ANOVA. YM201636 decreased PtdIns5P and PtdIns(3,5)P_2_ and both remained similarly reduced by pretreatment with BafA1. PtdIns3P increased by ~1.6-fold above control levels by YM201636 but only ~1.15-fold after pretreating with BafA1. (*), *P*<0.05.

YM201636, the first compound characterized as a specific PIKfyve inhibitor, has been widely used as a probe to uncover various cellular functions of PIKfyve [8, 9, 52]. It inhibits PtdIns(3,5)P_2_ and PtdIns5P synthesis with an IC_50_ of 33 nM and 25 nM, respectively [20, 37]. Whereas we have previously demonstrated that short pretreatment with low doses of BafA1 (15 nM, 40 min) rendered COS7 cells resistant to cytoplasmic vacuolization upon subsequent PIKfyve inhibition by YM201636 [30], the PI profiles remained to be examined. Moreover, in the light of recent observations for different functional outcomes of YM201636 and apilimod despite that they both inhibited specifically PIKfyve and triggered vacuole formation, such analysis is warranted [38]. Therefore, to quantify PIs and reveal whether the prevented PtdIns3P rise by BafA1 pretreatment could be reproduced with the YM201636 compound, we performed similar *myo*-[2-^3^H]inositol labeling experiments in HEK293 cells. At the end of the labeling protocol, cells were first treated with BafA1 (15 nM, 40 min) followed by YM201636 (800 nM, 60 min). Lipids were then extracted, deacylated and analyzed by HPLC. As illustrated in Fig 6, YM201636 reduced steady-state levels of PtdIns(3,5)P_2_ and PtdIns5P and they both remained equally reduced following BafA1 treatment. Conspicuously, however, whereas steady-state levels of PtdIns3P increased ~1.6-fold above controls by YM201636, this rise was only by ~1.15-fold if BafA1 was added prior to YM201636 (Fig 6). Collectively, these data unequivocally demonstrate that BafA1 prevention of cell vacuolization coincides with suppressed PtdIns3P elevation under PIKfyve inhibition by either apilimod or YM201636.

### Opposite effects of apilimod vs. BafA1/apilimod on EEA1 membrane association

**Fig 7:**
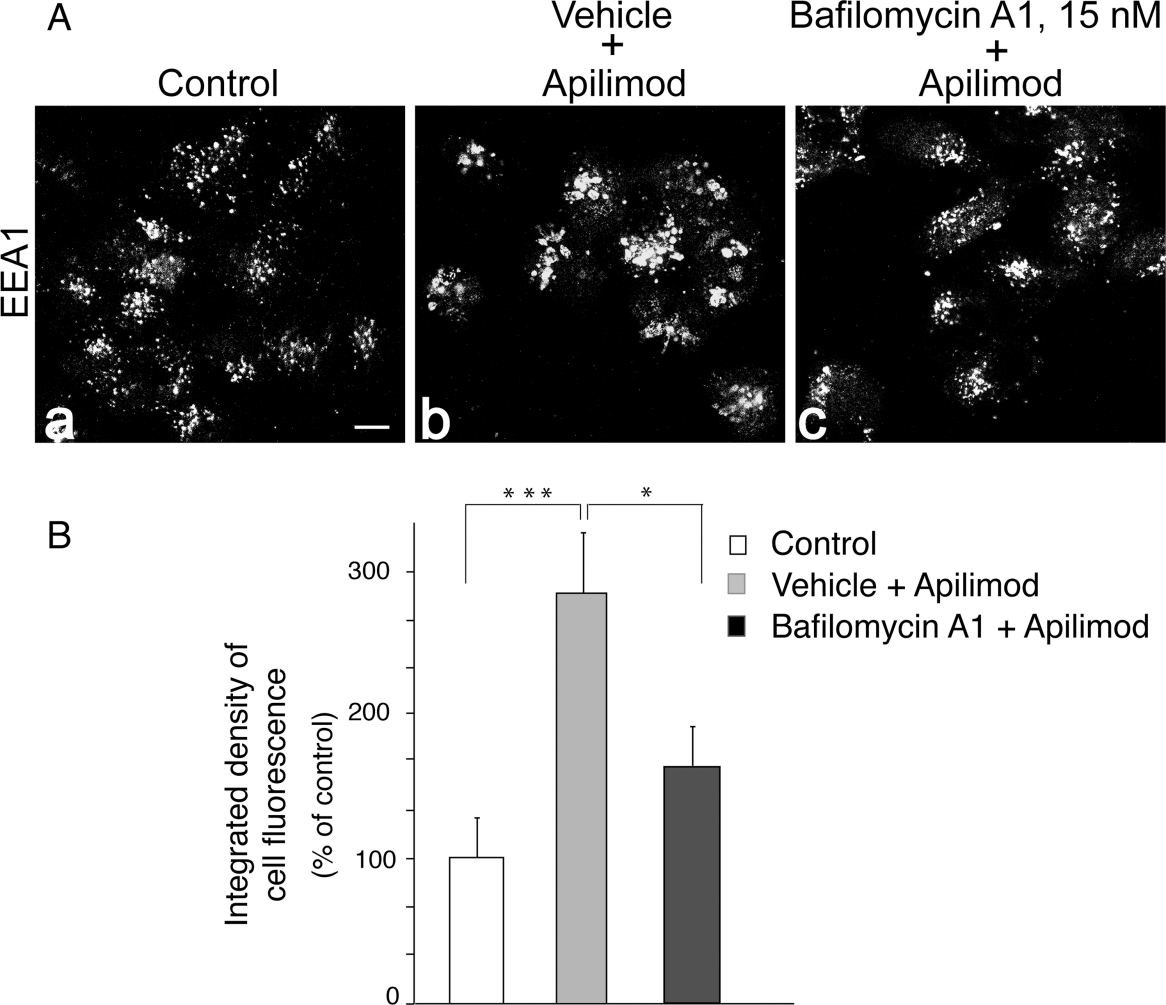
BafA1 precludes EEA1 membrane recruitment induced by PIKfyve inhibition with apilimod. HEK293 cells were treated with vehicle (0.1% DMSO, panels a and b) or BafA1 (15 nM) for 40 min followed by 100 nM apilimod (or 0.1% DMSO in the control) for an additional 60 min. Cells were then fixed, permeabilized, immunostained for EEA1 and observed by confocal microscope (40x objective). (A): Shown are typical immunofluorescence images for EEA1 (panels a—c) illustrating that fluorescence signals are markedly increased in cells with apilimod treatment (panels b vs. a) and dramatically diminished upon BafA1 pretreatment (panels b vs. c). (B): quantitation of the EEA1-associated immunofluorescence by ImageJ software based on randomly selected cells (30 cells/condition) from different fields in 2 separate experiments with similar results. Data are expressed as corrected integrated density of cell fluorescence (mean ± SEM) and analyzed by one-way Anova, *P<0.05; *** *P*<0.001. Bar, 10 μm.

Our observations for hampered increases of PtdIns3P (Figs 4 and 5) suggest that PtdIns3P down-regulation by BafA1 might be a new mechanistic determinant underlying the abolished cytoplasmic vacuolization despite PIKfyve inhibition. PtdIns3P is primarily distributed in the endosomal system [53]. It plays a crucial role in the homotypic early endosomes fusion by recruiting the cytosolic EEA1 protein [53]. Therefore, we surmised that BafA1’s ability to prevent cytoplasmic vacuolization under PIKfyve inhibition is due to reduced endosome recruitment of EEA1 as a result of dampened endosomal PtdIns3P. To test this, we performed immunofluorescence microscopy analyses of endogenous EEA1 in HEK293 cells that were treated with BafA1 prior to apilimod, *i*.*e*., a condition that prevents not only PtdIns3P rise but also the cytoplasmic vacuolization (see Figs 3 and 4). As illustrated in Fig 7A under apilimod treatment and, hence, elevated PtdIns3P and vacuolation (see Figs 2 and 3), we observed marked increases in the membrane-associated EEA1 fluorescence signals vs. those in control vehicle-treated cells. By contrast, cells with suppressed PtdIns3P elevation due to BafA1 displayed significantly reduced membrane-associated EEA1 fluorescence signals in parallel with the restored endomembrane morphology (Fig 7A). Quantitation of the fluorescence by ImageJ software from 30 randomly selected cells under each condition indicated that the rise of membrane-associated EEA1 immunofluorescence by apilimod treatment was 2.7-fold over the control, whereas that by BafA1/apilimod treatment was only ~1.7-fold (Fig 7B). Together, these data demonstrate reduced EEA1 endosome recruitment under BafA1-dependent suppression of both PtdIns3P rise and the cytoplasmic vacuolization.

## Discussion

Data for early embryonic lethality of the systemic *pikfyve* knockout in mice has unveiled the critical role of PIKfyve in cell proliferation and viability [19, 54, 55]. Consequently, several PIKfyve inhibitors have been found to exhibit anti-proliferative capacity and cytotoxicity, bringing to light a potential new modality in anti-cancer therapy [27, 56]. Among those, apilimod appears to be the most promising and is currently in clinical trials as an anti-cancer drug [28] yet its potential as a PIKfyve inhibitor remains only partially characterized. In this study we took advantage of our ability to unambiguously quantify alterations of the two PIKfyve lipid products *in vitro* and in a cellular context. We report for the first time that apilimod powerfully inhibits PIKfyve kinase activity not only for PtdIns(3,5)P_2_ but also for PtdIns5P synthesis. The IC_50_ for both products was estimated to be in the subnanomolar range (Fig 1). Furthermore, both PtdIns(3,5)P_2_ and PtdIns5P lipid products were markedly and similarly reduced in different cell lines treated with 100 nM apilimod (Figs 2 and 3). These new data provide clarification of the apilimod inhibitory potency and might further enlighten the molecular basis of its usage in cancer therapy.

As common for PIKfyve inhibitors, apilimod application in cells also triggers appearance of cytoplasmic vacuoles ([20, 22, 57, 58] and this study). Although the aberrant vacuolization conundrum seen upon PIKfyve perturbations has been widely used as a sensitive functional measure of localized PtdIns(3,5)P_2_ reduction, we demonstrate herein that the latter is necessary but not sufficient to trigger appearance of vacuoles under PIKfyve inhibition. Thus, we unraveled that the cytoplasmic vacuolization was precluded or was readily reversed by BafA1 in cells treated with apilimod yet alleviation of reduced PtdIns(3,5)P_2_ levels did not occur (Figs 4 and 5). Our observations for suppressed PtdIns3P surge by BafA1 suggest that not only the PtdIns(3,5)P_2_ reduction but also the concomitant PtdIns3P elevation are required to induce cytoplasmic vacuolization. The BafA1-dependent suppression of the PtdIns3P rise, documented in our study by both HPLC and a PtdIns3P reporter (Fig 5) was unconditional and was apparent not only upon PIKfyve inhibition by apilimod but also by the widely used YM201636 compound (Fig 6). Thus, our data imply that whereas PtdIns(3,5)P_2_ reduction under PIKfyve inhibition is a necessary it is not a sufficient condition for manifestation of the vacuolation phenotype. Rather, a concomitant rise in PtdIns3P that occurs due to arrested PtdIns3P consumption for PtdIns(3,5)P_2_ synthesis under PIKfyve perturbation [19, 22, 37, 48] should also be present.

PtdIns3P is primarily distributed in the endosomal system [53]. It not only serves as a substrate for PIKfyve catalyzed PtdIns(3,5)P_2_ production but also plays a central role in the homotypic early endosome fusion by recruiting the fusogenic EEA1 protein [53]. Recruitment of EEA1 to PtdIns3P as well as to active Rab5GTP is an essential step in endosome membrane tethering and fusion and the subsequent interactions with endosomal SNARE proteins [53, 59–62]. Our previous studies utilizing an established reconstitution assay for endosome fusion under conditions of enhanced or perturbed endogenous PIKfyve enzymatic activity, and hence, reduced or elevated cellular PtdIns3P, respectively, uncovered that PIKfyve is a powerful negative regulator of endosome fusion [44]. Concordantly, elevation of PtdIns3P through extensive increases of the Sac3 phosphatase, the PIKfyve-associated antagonistic enzyme that turns over PtdIns(3,5)P_2_ to PtdIns3P, was associated with a striking enhancement of EEA1 distributed on the dilated endosomes [16]. Our data herein for suppressed PtdIns3P elevation and decreased recruitment of the fusogenic EEA1protein (Figs 5–7) provide a mechanistic basis of the precluded cytoplasmic vacuolization by BafA1. Thus, BafA1 counterbalances the intensified fusogenic activity under PIKfyve inhibition by reducing the PtdIns3P rise.

One point that requires further investigation is the mechanism whereby BafA1 precludes the PtdIns3P rise under PIKfyve inhibition. Endosomal PtdIns3P is produced by the evolutionarily conserved Vps34 that forms a hetero-oligomeric complex with the putative protein kinase Vps15 and several accessory subunits [53, 63]. Vps15 is essential for Vps34 localization as it targets the kinase to endosomal membranes through recruitment onto active Rab5GTP. Additionally, a member of the myotubularin phosphatases (MTM1) that turn over PtdIns3P, was found to associate with Vps15/Vps34 and Rab5GTP [64]. Thus, BafA1 could compromise proper performance of each of the above-listed or yet-to-be identified steps as well as of their coordination in time and space to account for the suppressed PtdIns3P elevation under PIKfyve inhibition. Whereas the contribution for each of these events in the BafA1 rescue under PIKfyve inhibition should be addressed in future studies, it is intriguing that BafA1 also reverses or prevents the cytoplasmic vacuolization under Vps34 perturbations that markedly reduce the PtdIns3P pool along with that of PtdIns(3,5)P_2_ [30, 40]. It is conceivable that under the above or other vacuolation conditions [65, 66], BafA1-triggered rescue may engage other pH-dependent or–independent endosomal events or effectors that regulate endocytic traffic progression [67]. Those may include an arrest of the GTP-GDP functional cycle of Rab5 [30], attenuated recruitment of Arf6 nucleotide exchange factor ARNO to early endosomes [68] or yet-to-be-identified mechanisms that might emerge as research in the field progresses.

## Conclusions

Our study characterizes for the first time that apilimod inhibits not only PIKfyve- catalyzed PtdIns(3,5)P_2_ but also PtdIns5P production and reveals that the BafA1 rescue of the endomembrane vacuolation triggered by the drug is mechanistically associated with attenuated PtdIns3P elevation and suppressed recruitment of fusogenic EEA1. In addition to basic impact, the data in this study have also clinical relevance. For example, if apilimod is approved as a drug in cancer treatment, cautions should be taken in combined therapy with medications that affect PtdIns3P levels as such conditions might impair apilimod cytotoxicity.

## Supporting information

S1 Fig Cell treatment with only BAfA1 does not alter PtdIns3P. (A and B): HEK293 cells cultured in complete media grown to 90–100% confluence run in parallel with apilimod and BafA1+apilimod samples shown in Fig 5 were labeled for 24 h with 25 μCi/mL *myo*-[2-^3^H]inositol. Cells were than treated in the same labeling medium with vehicle (control, 0.1% DMSO) or BafA1 (15 nM in DMSO) for 40 min when additional vehicle (0.1% DMSO) was added for 60 min. Lipids were extracted, deacylated and subjected to HPLC separation. Shown are representative HPLC [^3^H]GroPInsP profiles from control (left panel) and BafA1-treated (right panel) cells (A) and quantification of bafilomycin-dependent changes in steady-state levels of PtdIns3P, PtdIns4P, PtdIns5P, PtdIns(3,5)P_2_ and PtdIns(4,5)P_2_ from 3 independent experiments (B). Apparent is the lack of changes in steady-state levels of PtdIns3P, PtdIns4P, PtdIns(3,5)P_2_ and PtdIns(4,5)P_2_ by BafA1. For reasons that remain to be clarified in future studies, there was a 40% decrease in steady-state levels of PtdIns5P compared to the control that received only vehicle. Note that BafA1 did not alter low levels of PtdIns5P reduced upon apilimod treatment (see Fig 5A and 5B).
(PDF)
