# Supplementary material for: Apilimod, a candidate anticancer therapeutic, arrests not only PtdIns(3,5)P2 but also PtdIns5P synthesis by PIKfyve and induces bafilomycin A1-reversible aberrant endomembrane dilation
Source: PLoS One. 2018 Sep 21;13(9):e0204532. doi: 10.1371/journal.pone.0204532 (PMC6150535; doi:10.1371/journal.pone.0204532)
Supplement: S1 Fig — (A and B): HEK293 cells cultured in complete media grown to 90–100% confluence run in parallel with apilimod and BafA1+apilimod samples shown in Fig 5 were labeled for 24 h with 25 μCi/mL myo-[2-3H]inositol. Cells were than treated in the same labeling medium with vehicle (control, 0.1% DMSO) or BafA1 (15 nM in DMSO) for 40 min when additional vehicle (0.1% DMSO) was added for 60 min. Lipids were extracted, deacylated and subjected to HPLC separation. Shown are representative HPLC [3H]GroPInsP profiles from control (left panel) and BafA1-treated (right panel) cells (A) and quantification of bafilomycin-dependent changes in steady-state levels of PtdIns3P, PtdIns4P, PtdIns5P, PtdIns(3,5)P2 and PtdIns(4,5)P2 from 3 independent experiments (B). Apparent is the lack of changes in steady-state levels of PtdIns3P, PtdIns4P, PtdIns(3,5)P2 and PtdIns(4,5)P2 by BafA1. For reasons that remain to be clarified in future studies, there was a 40% decrease in steady-state levels of PtdIns5P compared to the control that received only vehicle. Note that BafA1 did not alter low levels of PtdIns5P reduced upon apilimod treatment (see Fig 5A and 5B). (PDF) [file pone.0204532.s001.pdf]

Supporting Information

S1 Figure

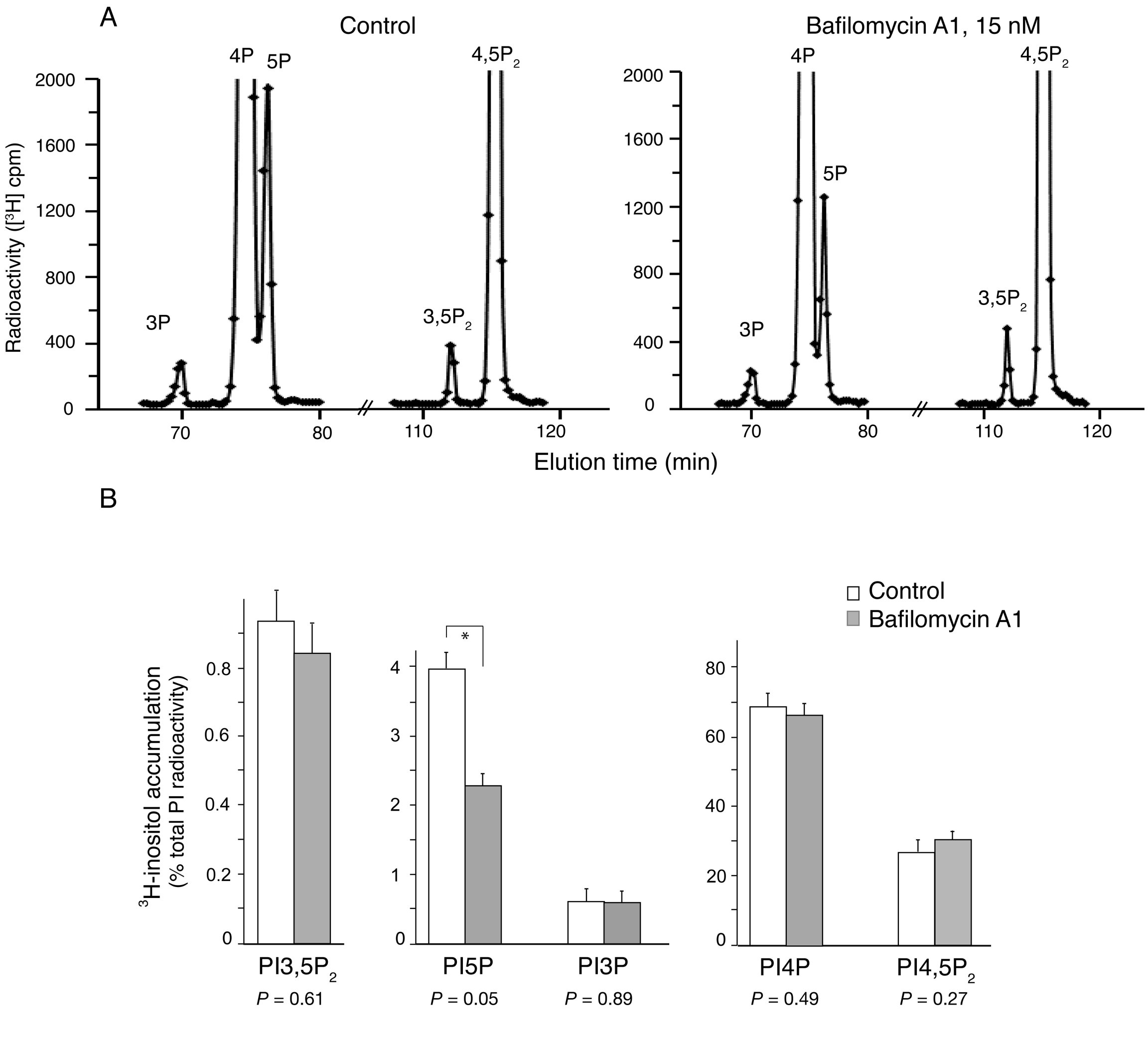

Supporting Information Caption

S1. Cell treatment with only BAfA1 does not alter PtdIns3P. (A and B): HEK293 cells cultured in complete media grown to 90-100% confluence run in parallel with apilimod and BafA1+apilimod samples shown in Fig 5 were labeled for 25 h with 25 μCi/mL *myo*-[2-^{3}H]inositol. Cells were than treated in the same labeling medium with vehicle (control, 0.1% DMSO) or BafA1 (15 nM in DMSO) for 40 min when additional vehicle (0.1% DMSO) was added for 60 min. Lipids were extracted, deacylated and subjected to HPLC separation. Shown are representative HPLC [^{3}H]GroPInsP profiles from control (left panel) and BafA1-treated (right panel) cells (A) and quantification of bafilomycin-dependent changes in steady-state levels of PtdIns3P, PtdIns4P, PtdIns5P, PtdIns(3,5)P_2 and PtdIns(4,5)P_2 from 3 independent experiments (B). Apparent is the lack of changes in steady-state levels of PtdIns3P, PtdIns4P, PtdIns(3,5)P_2 and PtdIns(4,5)P_2 by BafA1. For reasons that remain to be clarified in future studies, there was a 40% decrease in steady-state levels of PtdIns5P compared to the control that received only vehicle. Note that BafA1 did not alter low levels of PtdIns5P reduced upon apilimod treatment (see Fig 5AB).
